# Tailoring porosity and surface functionalization in nanoporous anodic alumina rugate filters for enhanced interferometric biosensing

**DOI:** 10.1039/d6na00533k

**Published:** 2026-09-02

**Authors:** Josep Maria Cantons, Pilar Formentin, Josep Ferré-Borrull, Akash Bachhuka, Lluis F. Marsal

**Affiliations:** a Department of Electronics, Electric and Automatic Engineering, Rovira i Virgili University (URV) 43007 Tarragona Spain lluis.marsal@urv.cat; b Institute of Chemical Research of Catalonia (ICIQ) 43007 Tarragona Spain

## Abstract

The influence of porosity and surface functionalization strategies on the biosensing performance of nanoporous anodic alumina rugate filters (NAA-RFs) is systematically investigated using reflectometric interference spectroscopy (RIfS). Highly ordered NAA-RF structures were fabricated by sinusoidal anodization and subsequently tuned through controlled pore-widening processes to achieve different porosity levels. Structural and optical characterization confirmed the formation of modulated nanoporous architectures exhibiting well-defined Fabry–Pérot interference patterns and characteristic stop bands, whose spectral position depends on the effective refractive index of the structure. The sensing performance was evaluated using two distinct surface functionalization strategies. A sequential electrostatic approach based on protein A (SpA) and anti-IgG was employed for IgG detection, while a covalent functionalization using APTES and glutaraldehyde (GTA) enabled the detection of lactate dehydrogenase (LDH). The results demonstrate that increasing porosity significantly enhances the effective optical thickness (EOT) response due to the larger internal surface area available for molecular binding. Kinetic analysis further revealed that higher porosity leads to not only increased saturation amplitudes but also longer characteristic response times, indicating diffusion limitations within the nanoporous network. Overall, the results highlight the importance of simultaneously tailoring structural parameters and surface chemistry to optimize the performance of NAA-based interferometric biosensors, demonstrating the potential of modulated nanoporous architectures for sensitive and selective biomolecule detection.

## Abstract 2

The influence of porosity and surface functionalization strategies on the biosensing performance of nanoporous anodic alumina rugate filters (NAA-RFs) is systematically investigated using reflectometric interference spectroscopy (RIfS).

## Graphical abstract

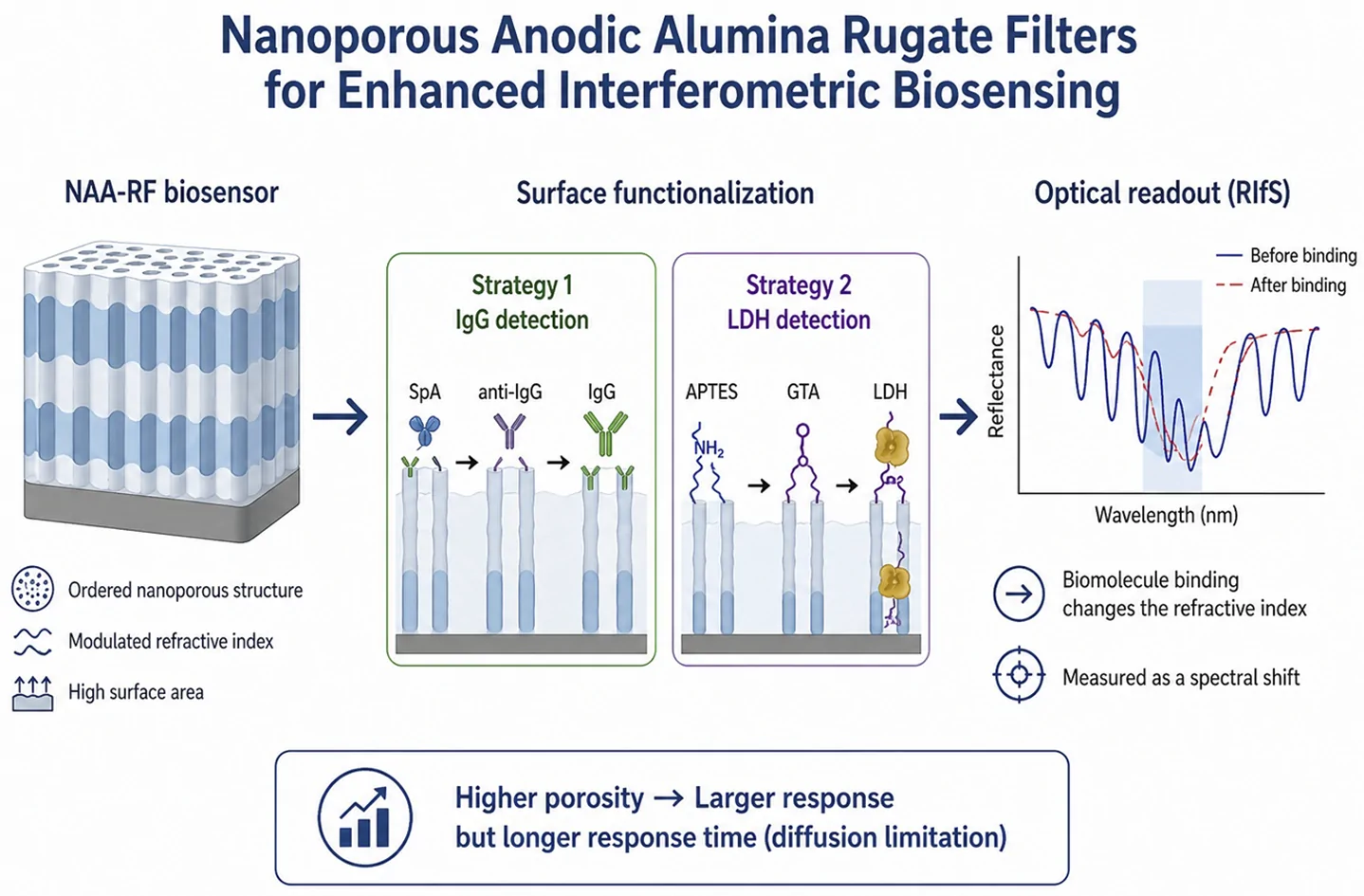

## 1. Introduction

Biosensing technologies have gained significant attention in recent years due to their ability to provide rapid, sensitive, and accurate detection of disease-related biomarkers.^1,2^ These platforms are increasingly recognized as key tools for improving healthcare accessibility by enabling cost-effective and decentralized diagnostic approaches. In contrast to conventional laboratory-based assays, biosensors offer rapid analysis and point-of-care capabilities, which are particularly valuable for early disease detection and continuous monitoring of patient conditions.^3^ Moreover, biosensors support the development of personalized medicine by providing real-time molecular information that facilitates biomarker analysis, patient stratification, and informed clinical decision-making.^4,5^ The growing demand for the simultaneous detection of multiple biomarkers, especially in complex diseases such as cancer and cardiovascular disorders, has further driven the development of advanced biosensing platforms capable of high-throughput and reliable measurements.^6,7^ Consequently, improving key sensing parameters, including sensitivity, limit of detection (LOD), specificity, and reproducibility, remains essential for expanding the applicability of biosensors in clinical diagnostics.

In recent years, porous materials have gained attention in the development of biosensors due to their remarkable physicochemical properties. Characteristics such as high surface area together with tunable pore size and geometry enable these materials to significantly enhance key sensing parameters involved in biomolecule detection.^8^ According to the International Union of Pure and Applied Chemistry (IUPAC) recommendations for the characterization of porous solids, porous materials are commonly classified according to their pore dimensions as microporous (<2 nm), mesoporous (2–50 nm), and macroporous (>50 nm).^9,10^ Compared with bulk materials, porous architectures provide several advantages, including improved analyte enrichment, molecular confinement, and enhanced signal.^11^ These features make porous materials highly attractive for transforming conventional, labor-intensive diagnostic methods into efficient and high-performance sensing platforms suitable for clinical applications. Among the porous materials explored for biosensing, silica-based porous materials,^12–16^ porous carbon materials,^17^ and nanoporous anodic alumina (NAA) have been widely investigated.^18–21^

NAA, obtained through the electrochemical anodization of aluminum, has emerged as a promising platform for optical sensing applications.^22–24^ This material provides several advantageous features, including tunable nanopore geometry, the possibility of surface functionalization to tailor the selectivity toward specific biomolecules, stable optical responses, and good biocompatibility.^25^ In addition, the anodization parameters can be carefully controlled to produce modulated nanostructures such as NAA rugate filters (NAA-RFs). These structures are formed by periodically varying the anodization conditions, generating a porous layer in which the effective refractive index is sinusoidally modulated along the depth of the film.^26–28^ The highly ordered architecture of NAA, together with the precise control over structural parameters such as pore diameter, porosity, and modulation period, makes this material particularly suitable for the fabrication of interferometric sensing platforms based on reflectometric interference spectroscopy (RIfS).^29^

These porous materials can be functionalized using a variety of surface chemistry strategies to render the inner surface selective toward specific target molecules.^30^ Different functionalization approaches have been reported to tailor the chemical properties of nanoporous structures, including silanization, polymer grafting, and biomolecule immobilization.^31^ Such modifications enable the introduction of functional groups capable of selectively interacting with target analytes, thereby improving the specificity and performance of the sensing platform.^32^ In the case of nanoporous anodic alumina, surface functionalization is particularly advantageous due to the large internal surface area of the nanopores, which provides numerous active sites for molecular attachment and recognition events.^33^

In this study, NAA-RF nanostructures were used to evaluate two different surface functionalization strategies for the selective detection of two target biomolecules. The first approach is based on the electrostatic immobilization of protein A (SpA) onto the inner pore surface, which enables the subsequent binding of anti-IgG antibodies for the detection of IgG antigens. The second strategy relies on chemical functionalization of the nanopores using APTES and glutaraldehyde (GTA), allowing the immobilization and detection of lactate dehydrogenase (LDH). These approaches demonstrate how different surface chemistry methods can be applied to tailor the selectivity of NAA-RF structures toward specific biomolecular targets. Another objective of this work is to improve the sensing performance by exploiting the modulated architecture of NAA-RFs, which can provide higher sensitivity compared with structures containing straight nanopores.^34^ This is because of the modulated pore diameter in depth of NAA modulated structures, which creates a periodically modulated effective refractive index. This design also provides more effective surface area due to the periodical structure which enhances the possibility of infiltration of a higher number of biomolecules in the inner structure and by consequence having a more sensitive biosensor.^35,36^ In this study we use reflectometric interference spectroscopy (RIfS) as a promising technique to study the modulated nanostructures for biosensing applications. RIfS is based on the interaction of broadband light with a solid-state thin film, generating an interference pattern associated with the Fabry–Pérot interference pattern.^37^ NAA, fabricated by aluminum anodization, represents an excellent platform for RIfS-based sensing due to its tunable nanoporous geometry and the possibility of surface functionalization to achieve selective detection of target biomolecules. In this work, we investigate two different types of functionalization of NAA-RFs with different porosities in order to evaluate their effective optical properties. This is achieved through controlled infiltration of different solutions, enabling the estimation of porosity and its influence on the optical response. Furthermore, the sensing performance of NAA-RF structures is evaluated using a sequential functionalization strategy involving SpA, anti-IgG, and IgG, while LDH detection is performed using an alternative surface chemistry method. These experiments allow us to study the effect of porosity on the optical response and to evaluate the potential of these structures for enhancing sensing performance within two different surface chemistry methods.

## 2. Experimental section

### 2.1. Materials

High-purity aluminium substrates (99.999%) with a thickness of 0.5 mm were purchased from Goodfellow Cambridge Ltd (UK). Ethanol (C_2_H_6_O), acetone (C_3_H_6_O), oxalic acid (C_2_H_2_O_4_), perchloric acid (HClO_4_), chromic acid (H_2_CrO_4_), 3-aminopropyltriethoxysilane (C_9_H_23_NO_3_Si, APTES), hydrogen peroxide (H_2_O_2_), glutaraldehyde (CH_2_(CH_2_CHO)_2_, GTA), l-lactate dehydrogenase (LDH) (150 kDa), SpA (42 kDa), anti-IgG (150 kDa), and IgG (150 kDa) were provided by Sigma-Aldrich. Double de-ionized (DI) water (18.6 MΩ, Milli-Q®) was used for all the solutions unless otherwise specified.

### 2.2. Fabrication of nanoporous anodic alumina

**Fig. 1:**
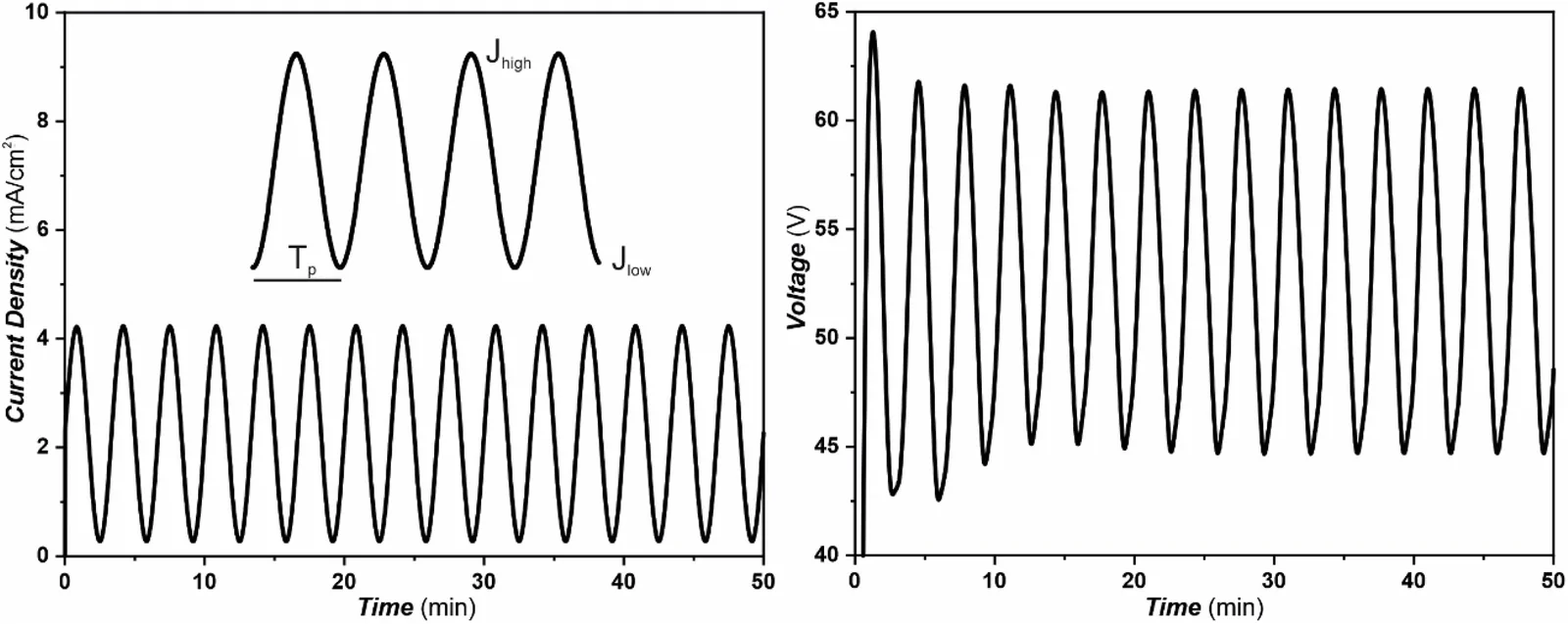
Anodization current density profile and voltage profile of the NAA-RF. The left image shows the sinusoidal current density profile of the NAA-RF and the inset shows 4 of the 38 periods that go from *J*_low_ to *J*_high_, with *T*_p_ being the time duration of a period. The right image shows the voltage profile that is produced while applying the sinusoidal current density.

Nanoporous anodic alumina (NAA) films were fabricated on 2 × 2 cm aluminum substrates through a two-step anodization process. Prior to anodization, the aluminum substrates were sequentially cleaned in acetone, Milli-Q water, and ethanol under ultrasonication for 5 min each to remove organic residues and surface contaminants. The cleaned substrates were then electropolished in an ethanol/perchloric acid solution (EtOH : HClO_4_, 4 : 1 v/v) at 20 V for 6 min to obtain a smooth and mirror-like aluminum surface. The first anodization step was carried out in 0.3 M oxalic acid (H_2_C_2_O_4_) at 40 V and 5 °C for 20 h. This process generated a porous alumina layer with a partially disordered pore arrangement at the top surface, while a progressive mechano-electrochemical self-ordering mechanism occurred at the alumina/aluminum interface. The as-formed alumina layer was subsequently removed by chemical etching in a mixture of 0.4 M H_3_PO_4_ and 0.2 M H_2_CrO_4_ at 40 °C for 24 h, resulting in the formation of a highly ordered array of nanoconcavities across the aluminum surface. Subsequently, the second anodization was performed using the same electrolyte conditions as in the first anodization step (0.3 M oxalic acid (H_2_C_2_O_4_) and at 5 °C). The anodization was then performed by applying an apodised current profile consisting on a sinusoidal component with variable current density amplitude in 1.77 cm^2^ of anodized area. The sinusoidal current profile applied during anodization was defined according to eqn (1):1
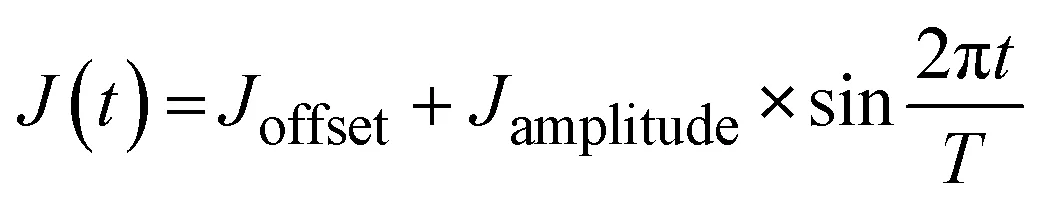
where *J*_offset_ is the current offset, *J*_amplitude_ is the current amplitude, and *T* is the period of the sinusoidal current profile. The amplitude of this AC component was modulated with a half-wave sinus profile with a *J*_high_ of 4.22 mA cm^−2^ of maximum amplitude and a *J*_low_ of 0.276 mA cm^−2^ of minimum amplitude.^38–41^ In Fig. 1 we observe the NAA-RF current density sinusoidal profile which shows 4 of the 38 periods applied that lasts 200 s per period. After anodization, pore widening was conducted by immersing the samples in 5 wt% H_3_PO_4_ at 35 °C for different time durations (5, 10, 15, 20 and 25 min) to increase the average pore diameter and the porosity. Finally, a thin gold layer was deposited on the top surface of the NAA-RF films by sputtering in order to enhance the optical response of the nanostructured platform.

### 2.3. Chemical functionalization of nanoporous anodic alumina

The inner surface of the as-produced NAA was first hydroxylated by immersion in hydrogen peroxide at 90 °C for 10 min. The hydroxylated samples were then chemically functionalized with 3-aminopropyltriethoxysilane (APTES) *via* chemical vapor deposition. The silanization process was carried out in a vacuum chamber at 110 °C for 2 h.^42–44^ Subsequently, the amino-functionalized NAA samples were incubated in a 2.5% (v/v) aqueous glutaraldehyde (GTA) solution for 1 h at room temperature to enable cross-linking.^45,46^ After incubation, the samples were thoroughly washed with deionized (DI) water to remove loosely bound GTA molecules.

### 2.4. Structural characterization of nanoporous anodic alumina

Morphological features were characterized using a Field Emission Scanning Electron Microscope (FESEM) (Scios 2, Thermo Fisher Scientific, Waltham, MA, USA) under a chamber pressure of 1 × 10^−4^ Pa, with electron beam voltages set between 30 kV and magnifications in a range from 50k× to 250k×, depending on the sample. The Everhart–Thornley detector (ETD) was used to detect secondary electrons (SEs).

### 2.5. Optical characterization of nanoporous anodic alumina

Reflection spectra of NAA-GPCs were recorded from 400 to 800 nm at 2 nm resolution and 8° incidence angle using a PerkinElmer UV-visible-NIR Lambda 950 spectrophotometer. Reflectance spectra of NAA interferometers were acquired by illuminating white light from a tungsten halogen lamp (Ocean Optics, LS-1) and recording the reflected light using an Ocean Optics spectrometer (USB2000). The collection of reflected light was performed by the optical-fiber probe and guided to the spectrometer, which recorded one spectrum every 30 s with an integration time of 100 ms and 10 average measurements. Reflectance spectra were measured for the wavelength range of 400–1000 nm. The resulting data were collected and EOT values were calculated by applying fast Fourier transform (FFT) to the collected spectra using IGOR Pro software from Wavemetrics Inc.

### 2.6. Surface sputtering

Gold was sputtered on the samples at 0.05 mbar and 30 mA for 30 s, resulting in approximately 8 nm of gold overlayer on the NAA, using an AGAR Auto Sputter Coater Aname (Madrid, Spain).

### 2.7. Reflectometric interference spectroscopy (RIfS) system

Reflectometric interference spectroscopy (RIfS) was used to track changes in the effective refractive index of the nanoporous anodic alumina (NAA) films resulting from molecular interactions occurring along the internal pore walls. The optical behavior of NAA strongly depends on its structural parameters, including pore geometry, film thickness, and material composition, which collectively determine its interference response upon illumination. When broadband light is incident on the porous layer, partial reflections occur at both the top and bottom interfaces of the NAA film. The superposition of these reflected beams generates an interference pattern characteristic of a Fabry–Pérot interference. The resulting reflectance spectra display periodic oscillations whose frequency is directly related to the effective optical thickness (EOT) of the layer.^47–50^ The EOT values were extracted by applying a Fourier transform to the reflectance spectra and can be described according to eqn (2).

2 EOT_eff_ = 2 × *n*_eff_ × *L*

where EOT_eff_ is the effective optical thickness, *n*_eff_ is the effective refractive index of the NAA, and *L* is the thickness of the nanostructure. The EOT peak is suitable for sensing applications, as molecular binding within the NAA modifies the effective medium, leading to shifts in the EOT_eff_ peak.^51–53^ To enhance the interference contrast, NAA surfaces were sputter-coated with a thin gold layer.

### 2.8. Sensing assessment of lactate dehydrogenase (LDH) and IgG antigen

Reflection spectra of NAA-based gradient photonic crystals (NAA-GPCs) were acquired in the 400–800 nm wavelength range with a spectral resolution of 2 nm and an incidence angle of 8° using a PerkinElmer Lambda 950 UV-vis-NIR spectrophotometer. Real-time optical measurements were performed under continuous flow conditions (25 µL min^−1^) using an acrylic flow cell. For NAA-RFs, reflectance spectra were collected using a halogen light source (LS-1LL, Ocean Optics, USA) coupled to a fiber-optic spectrometer. Illumination was directed onto the sample surface at normal incidence (0°) *via* a bifurcated fiber-optic probe composed of six peripheral illumination fibers surrounding a central collection fiber. An optical lens integrated into the probe focused the light onto an approximately 1 mm diameter circular spot on the NAA-RF surface. The reflected signal was guided back through the collection fiber to the spectrometer, which recorded spectra in the 400–1000 nm range at 1 s intervals with an integration time of 50 ms. Sensing experiments were conducted under dynamic flow conditions. For IgG detection, a three-step sequential binding protocol was implemented. First, protein A (SpA, 100 µg mL^−1^) was flowed to promote electrostatic adsorption onto the inner surface of the NAA-RF. Subsequently, anti-IgG antibodies (100 µg mL^−1^) were injected and allowed to bind to SpA through the Fc region for 90 min. Finally, IgG antigen (100 µg mL^−1^) was flowed through the system for 90 min to enable specific antigen–antibody recognition. Phosphate-buffered saline (PBS) was injected between each step to remove loosely bound molecules. For lactate dehydrogenase (LDH) sensing, a 100 µg per mL LDH solution was introduced into GTA-prefunctionalized NAA-RF samples, allowing covalent attachment through reaction with free aldehyde groups present on the inner pore surface. All recorded RIfS spectra were processed using fast Fourier transform (FFT) algorithms implemented in Igor Pro (Wavemetrics, USA) to extract the effective optical thickness (EOT) values.

## 3. Results and discussion

### 3.1. Structural characterization of NAA-RFs

**Fig. 2:**
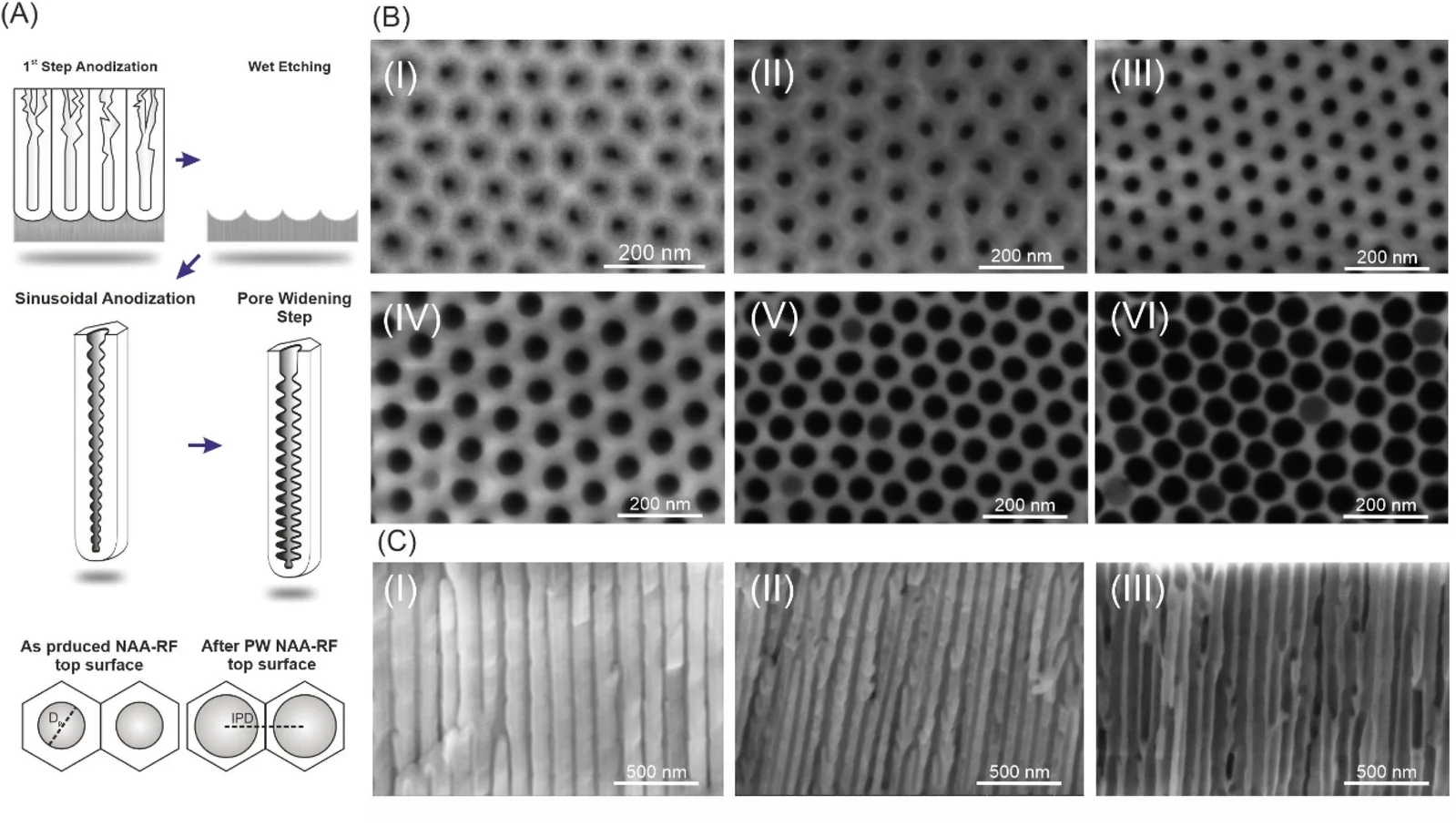
Fabrication schematic and structural characterization of NAA-RFs. (A) The schematic of the fabrication of NAA-RFs showing the two-step anodization (1st anodization, wet etching; 2nd step, anodization by sinusoidal current density) and pore widening reaction. (B) The FESEM top-view images: (I) the as fabricated NAA-RF, (II) after 5 minutes of pore widening (PW), (III) after 10 minutes of PW, (IV) after 15 minutes of PW, (V) after 20 minutes of PW, and (VI) after 25 minutes of PW. (C) The FESEM cross-sectional view: (I) the as fabricated NAA-RF, (II) after 10 minutes of PW, and (III) after 20 minnutes of PW.

Fig. 2A shows a schematic of the two-step anodization procedure where the resulting NAA-RF is observed after the sinusoidal anodization and how the structure after pore widening increases its surface area. These samples after fabrication were then characterized through FESEM imaging obtaining the morphological features of our structures. Fig. 2B shows the top-view images of the NAA-RF after different time durations of pore widening (0, 5, 10, 15, 20 and 25 min) from (I) to (VI), respectively. The hexagonal arrangement of the nanopores can be clearly seen. The images show pore sizes ranging from 24 ± 3 nm (Fig. 2B(I)) to 88 ± 4 nm (Fig. 2B(VI)), where nanopores are almost collapsing. Fig. 2C presents detailed cross-sectional view images of the NAA-RF showing how the width of the nanopores varies in depth for three different time durations of PW: (I) the as-produced NAA-RF, (II) NAA-RF after 15 minutes of PW and (III) after 25 minutes of PW. These modulations are a consequence of the applied sinusoidal current and in the three images it is observed that the layers corresponding to the high and low current density can be distinguished.

Table 1 summarizes the characteristic morphological values estimated from the FESEM characterization of NAA-RF top and cross-sectional views for each NAA-RF nanostructure. The included features are: the interpore distance (*D*_int_), pore diameter (*D*_p_) from top-view, pore diameter that corresponds to high current density (*D*_p_ (h)), pore diameter that corresponds to low current density (*D*_p_ (l)), and the physical period (*T*_p_) in depth. It can be observed how *D*_int_ is consistent after the different time durations of pore widening and also how the *T*_p_ is also not changing at all on each nanostructure. This *T*_p_ is a consequence of the applied sinusoidal current density and corresponds to one period of the modulated current. From these modulations we obtain two pore diameters that correspond to low and high current density. These pore diameters increase with the pore widening, resulting in a structure with a higher surface area when compared with a monolayer structure.

**Table 1:** Characteristic morphological values of NAA-RF samples for different pore widening times, including the interpore distance (*D*_int_), pore diameter from top-view (*D*_p_), pore diameters at high and low current density (*D*_p_ (h) and *D*_p_ (l)), and the physical period in depth (*T*_p_)

NAA-RF PW (min)	*D*_int_ (nm)	*D*_p_ (nm)	*D*_p_ (h) (nm)	*D*_p_ (l) (nm)	*T*_p_ (nm)
0	105 ± 3	24 ± 3	28 ± 3	22 ± 4	242 ± 4
5	108 ± 4	32 ± 2	37 ± 4	32 ± 3	235 ± 5
10	107 ± 2	42 ± 4	44 ± 3	38 ± 2	240 ± 6
15	107 ± 3	52 ± 4	54 ± 3	50 ± 2	230 ± 8
20	108 ± 3	72 ± 2	82 ± 2	74 ± 3	240 ± 5
25	108 ± 4	88 ± 4	98 ± 2	90 ± 4	245 ± 6

### 3.2. Optical characterization of NAA-RFs

**Fig. 3:**
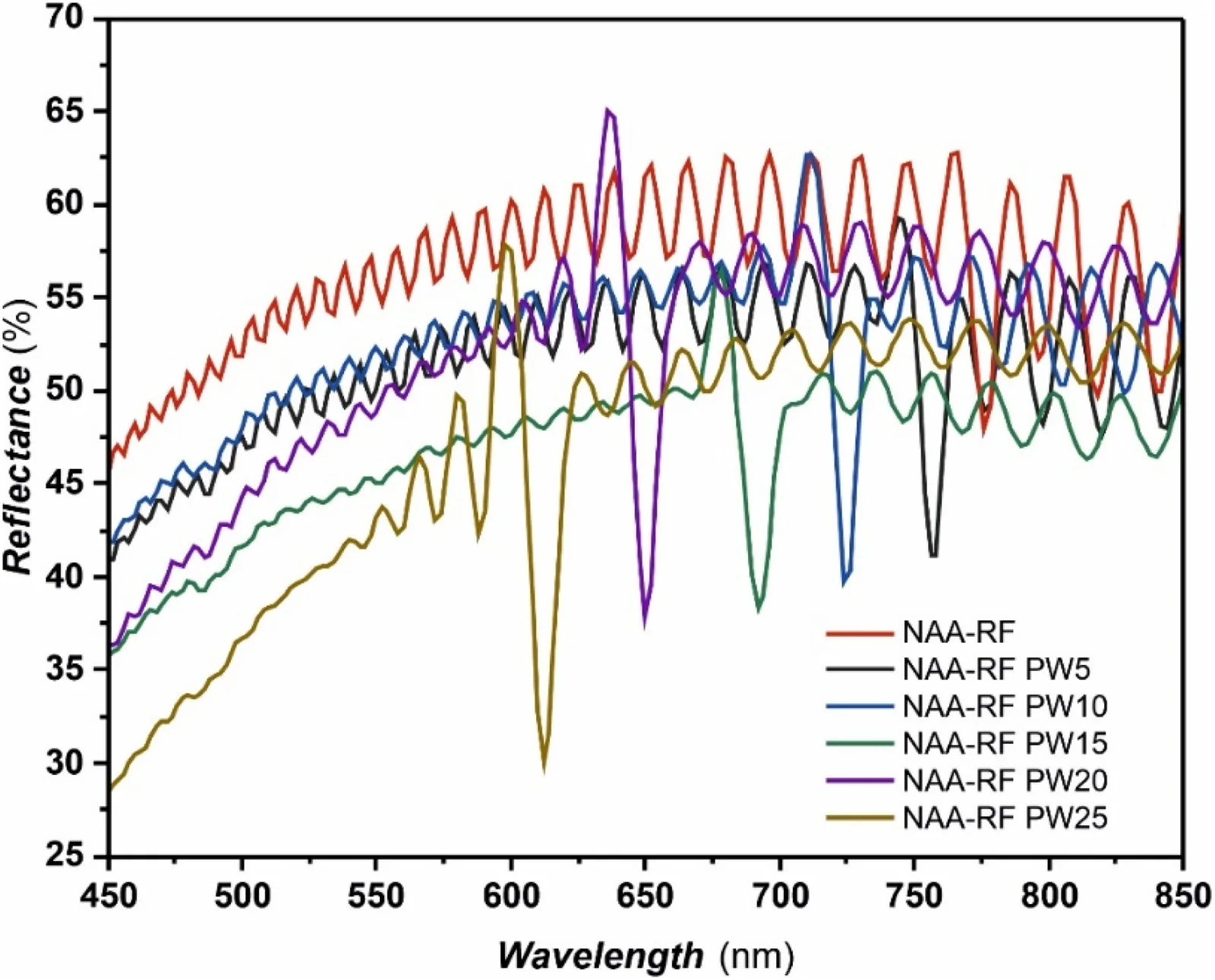
Interferometry spectra of NAA-RFs from the as fabricated to different applied time durations of pore widening showing the specific stop band position at a certain wavelength. The figure shows how the stop band shifts when increasing the pore widening time.

The modulated nanostructures were optically characterized through interferometric measurements. Fig. 3 shows the interference measurements of NAA-RFs after the fabrication process and the different applied PW times. The spectra show the reflectance response of NAA-RFs. In red we observe the as-produced samples showing oscillations through the visible range, known as Fabry–Pérot interferences. The spectra also show a characteristic stop band at a certain wavelength which is given by the modulated profile in the fabrication. The as-produced samples showed a stop band at 776 nm, and we can observe how this stop band shifts to the blue for each time duration of pore widening (5, 10, 15, 20, and 25 min). We obtained stop bands after pore widening at 756, 724, 692, 650, and 612 nm. The positions of the stop bands in NAA-RFs are determined by the optical path length of one period, which depends on the refractive indices of the layers formed under high and low anodization currents. The shift of the stop band we observe in NAA-RF structures happens as a result of the reduction of the effective refractive index of the structure when applying the pore widening. From the mentioned oscillations we can obtain the effective optical thickness of the NAA-RF structure which can be used for sensing purposes. Thanks to this and knowing the real thickness of each nanostructure we can calculate the effective refractive index of each of them and estimate their porosity Fig. S1.

### 3.3. Sensing assessment application of IgG

**Fig. 4:**
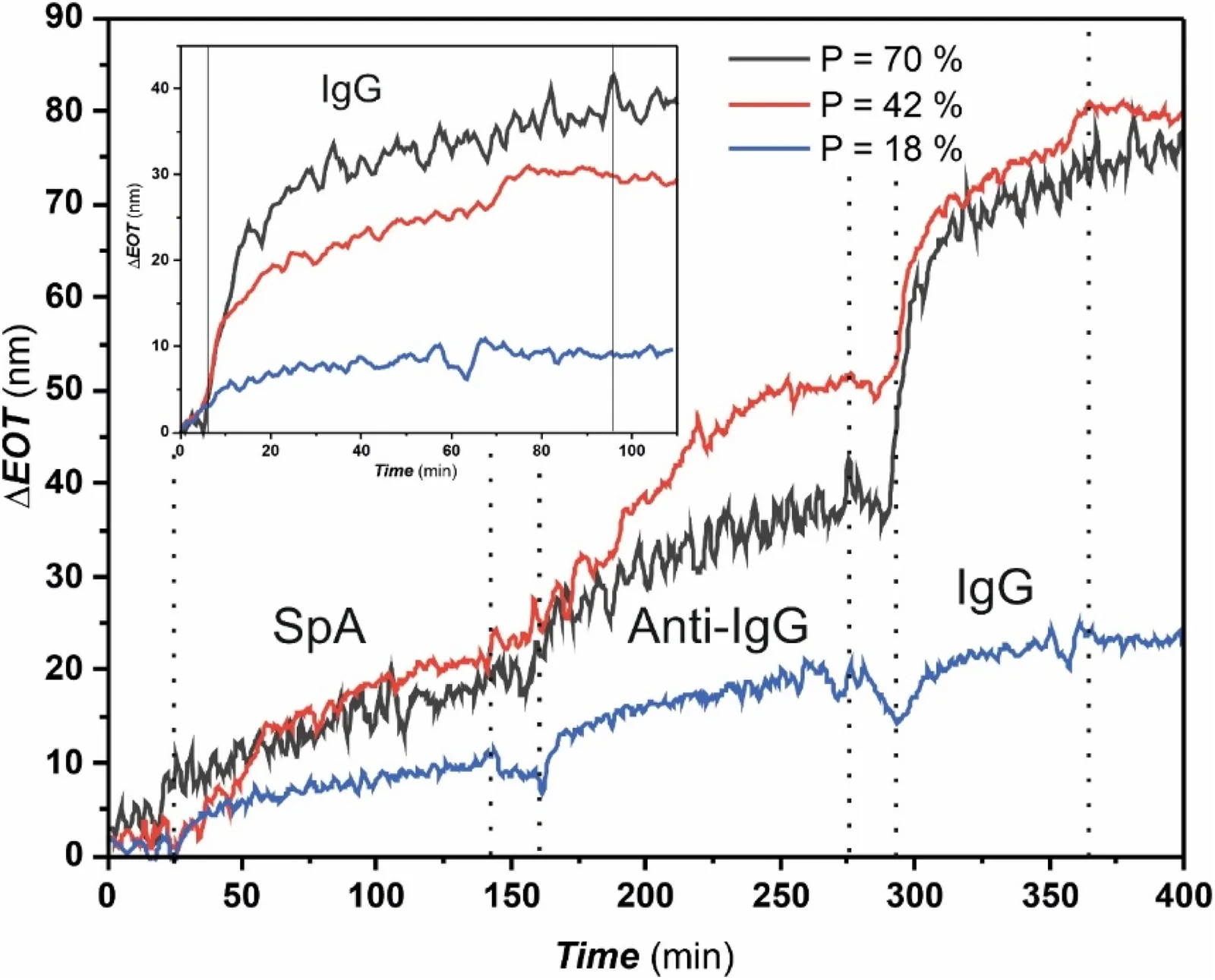
Real-time detection spectra of SpA, anti-IgG and IgG for NAA-RFs of different porosities showing how EOT shifts depending on the porosity. The inset shows the final detection of IgG with the real EOT shift after the binding of SpA and anti-IgG on the inner structure.

The non-functionalized NAA-RFs were then tested in a sensing experiment for the detection of IgG. This application aims to study the effect of porosity in terms of sensing by monitoring changes in the effective optical thickness. Three different porosities were considered to carry out the sensing experiments (18 ± 3, 42 ± 3 and 70 ± 4%). The experiments were performed following a sequential binding strategy. Initially, protein A (SpA) was introduced and electrostatically adsorbed onto the inner surface of the NAA-RF structure. Subsequently, anti-IgG antibodies were injected to bind specifically to the immobilized SpA. After stabilization of this intermediate layer, the target molecule (IgG) was introduced to allow antigen–antibody recognition. Fig. 4 shows the results of the sensing experiments for the three different porosities of NAA-RFs; they all show four intermediate washing steps with PBS to remove the non-bound molecules. For comparison purposes, the inset shows the ΔEOT of the IgG detection step for the three considered porosities starting at a common ΔEOT value referenced to a common PBS baseline. The blue curve corresponds to the sample with the lowest porosity (18%) where it can be observed how from the PBS baseline the EOT starts increasing when injecting the first protein (SpA) and the same happens for the antibody (anti-IgG). This effect is also observed for the 42% porosity (red line) and for the 70% porosity (black line). In this figure we can observe an increase in EOT for the three different stages of sensing. We obtained values for SpA detection of 10 ± 2 nm (18% porosity), 19 ± 3 nm (42% porosity) and 16 ± 2 nm (70% porosity). The least porous structure exhibited a lower response, whereas the two more porous structures showed similar changes. The same happens when injecting anti-IgG, where a ΔEOT of 11 ± 2 nm for the 18% porous NAA-RF, 27 ± 3 nm for 42% porosity and 25 ± 4 nm for 70% porosity was observed.

When injecting IgG we observed a less significant ΔEOT for the less porous NAA-RF whereas for the other two we found a high EOT response. For the 18% porosity NAA-RF, a ΔEOT of 7 ± 2 nm was obtained. This lower response may be attributed to the narrowest pore regions within the sinusoidal modulation profile, which can restrict IgG transport and become partially blocked after the previous immobilization of SpA and anti-IgG. In contrast, for the mid porous NAA-RF we find a ΔEOT of 29 ± 3 nm which is significantly higher than that for the lowest porosity NAA-RF. Moreover, in comparison, for the highest porosity NAA-RF (70% porosity) we can observe IgG signals up to ΔEOT of 42 ± 3 nm; this is due to the high difference in the porosity and in consequence, having a higher surface area than the low- and mid-porous NAA-RFs. Again, having higher ΔEOT for the two more porous structures is due to the higher effective surface area due to the large pore diameters obtained through the pore widening reactions and the modulated inner surface.

### 3.4. Sensing assessment of LDH

**Fig. 5:**
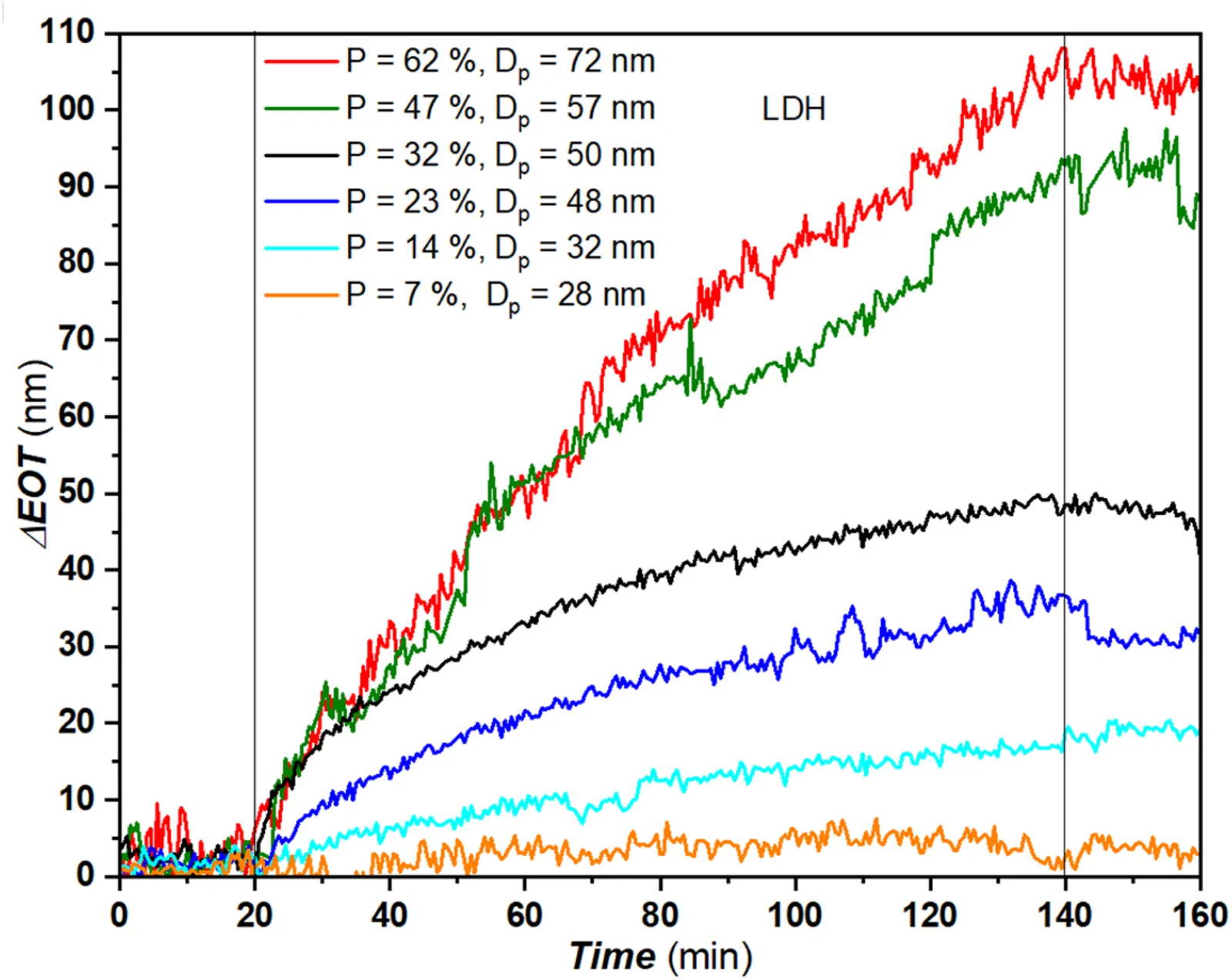
Real-time detection of LDH for NAA-RFs of different porosities showing the behavior of EOT when increasing the porosity.

The previously functionalized NAA-RF samples were subsequently evaluated in a separate real-time sensing experiment aimed at detecting LDH. In this case, LDH molecules form stable covalent bonds with the free aldehyde groups present on the GTA-modified inner pore surface of the NAA-RFs. To investigate the influence of structural parameters on sensing performance, six NAA-RF samples with different porosities were analyzed (7 ± 2, 14 ± 2, 23 ± 3, 32 ± 2, 47 ± 4, and 62 ± 4%). This approach allowed us to assess the effect of porosity on molecular binding efficiency and to demonstrate how alternative surface functionalization strategies can tailor the inner pore chemistry toward selective recognition of a specific biomolecule. Fig. 5 shows the real-time monitoring of 100 µg mL^−1^ of LDH through NAA-RFs of different porosities from a stable baseline of PBS and ending with a washing step of PBS to remove the non-bound molecules. For the less porous NAA-RF (7%) a non-significant change of 4 ± 2 nm can be observed; this changes when increasing the porosity to 14%, where we find values of ΔEOT of 19 ± 3 nm, which is significantly higher than the obtained signal for the less porous structure. These ΔEOT values increase for NAA-RFs with 23% and 32% porosity achieving values of ΔEOT of 36 ± 3 nm and 49 ± 2 nm, respectively, due to the increase in the porosity. This behavior is amplified when having porosities up to 47% and 62% where we obtained values of ΔEOT of 93 ± 3 nm and 108 ± 2 nm, respectively. This makes these samples the most sensitive structures when compared with the other 4 NAA-RF porosities. Focusing on the less porous structure (7%), the negligible response observed may result from restricted LDH transport through the narrowest pore regions, where the molecular dimensions of LDH are comparable to the available pore size. In contrast, for the highest porosity (62%) we observe the highest signal which is a consequence of this NAA-RF having a larger surface area. The higher response observed for the most porous NAA-RF may result from the combined effect of a larger accessible surface area and improved molecular transport within the nanoporous structure, whereas the lowest-porosity sample may experience partial pore blockage due to the comparable dimensions of the functional layer, LDH molecules, and the narrowest pore regions.

### 3.5. Kinetic analysis of LDH and IgG

**Fig. 6:**
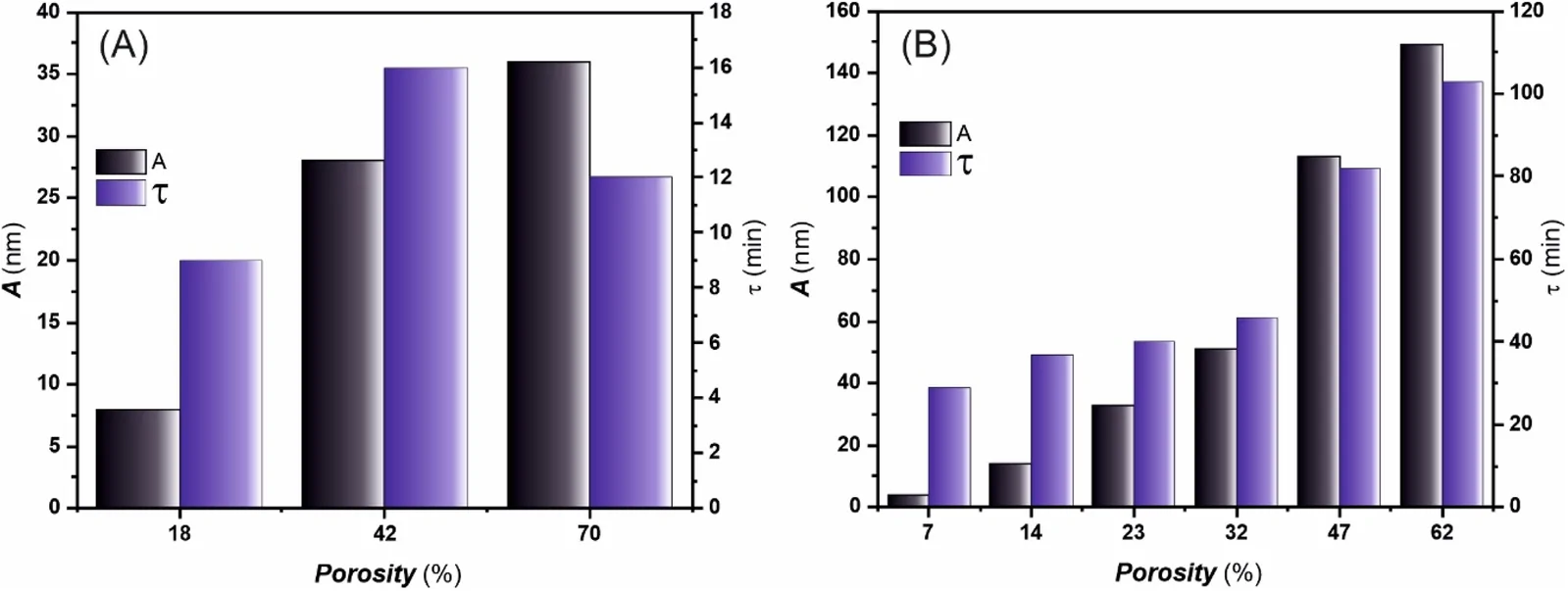
Summary of the kinetics study of IgG and LDH detection. (A) The behavior of the saturation amplitude and characteristic time for NAA-RFs of different porosities for IgG detection. (B) The behavior of the saturation constant and characteristic time for NAA-RFs of different porosities for LDH sensing.

The kinetic analysis was performed to assess how porosity influences the time-dependent sensing response and molecular transport within the NAA-RF structures. To study the binding kinetics of LDH and IgG, an exponential fitting was applied to the sensing experiments of both proteins. Fig. S2 presents the experimental ΔEOT evolution over time for both proteins at different porosities, together with the corresponding exponential fits (red curves), illustrating the agreement between the measured data and the applied kinetic model and enabling the extraction of the characteristic parameters for each condition. This fitting corresponds to the kinetic model which is defined as:3
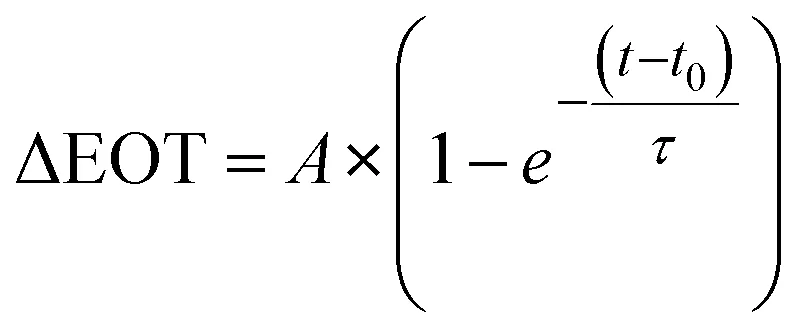
where *A* represents the saturation amplitude, *τ* is the characteristic response time of the binding process, and *t*_0_ is a reference time. The saturation amplitude is used as a quantitative measure of the response of the NAA-RFs for LDH and IgG detection. This approach enables the estimation of the binding kinetics of the NAA nanostructures with different porosities for the mentioned biomolecules. Fig. 6 shows a summary of the kinetics study representing the behavior of *A* and *τ* for IgG and LDH. Fig. 6A presents the kinetics study for IgG protein after the two previous binding steps of SpA and anti-IgG as a function of porosity. The behavior of *A* for IgG detection shows a clear increasing trend with porosity, with values of *A* = 8 nm for 18% porosity, *A* = 28 nm for 42% and *A* = 36 nm for 70% porosity. In terms of the characteristic response time no clear trend with porosity was observed, with values of *τ* = 9 for 18%, *τ* = 16 min for 42% and *τ* = 12 min for 70% with a slight decrease. Fig. 6B shows the behavior of *A* and *τ* for the detection of LDH for the different tested porosities (from 7 to 62%). Focusing on low and mid porosities we found low values of the saturation constant, obtaining values of ΔEOT of 4, 14 and 33 nm for porosities of 7, 14 and 23%, respectively. Instead, for structures with higher porosities, 47 and 62%, we found a significant increase in the saturation constant which varies from 41 to 149 nm. Observing the whole range of porosities, it is clearly seen that the saturation constant has a clear increasing tendency as observed with IgG detection. A similar behavior is observed for the characteristic time, which remains nearly constant from low to medium porosity, while a clear increase is observed at the highest porosity. For the first 4 porosities (7, 14, 23 and 32%) we obtained nearly constant values of *τ* of 29, 37, 40 and 46 minutes, respectively. This value of *τ* significantly increases for the NAA-RF with a porosity of 47% with a value of 82 minutes and achieving a value of 103 minutes for the NAA-RF with 62% porosity.

The results obtained for both IgG and LDH indicate that samples with higher porosity exhibit larger EOT saturation amplitudes. This behavior can be attributed to the increase in internal surface area associated with higher porosity, which provides a greater number of available binding sites along the inner pore walls, resulting in an enhanced optical response. Regarding the characteristic time constant, higher *τ* values can be linked to slower molecular transport within the porous matrix. In addition to molecular transport and binding-site availability, changes in pore diameter may also influence the wettability and liquid infiltration of the NAA-RF structures. Therefore, *τ* is mainly related to the time-dependent transport and binding processes, whereas *A* may reflect the combined effect of accessible binding sites, pore accessibility, and solution penetration within the nanoporous structure. For both proteins, the highest porosity samples exhibited larger *τ* values, suggesting that although increased porosity enhances the available binding surface, it may also lead to slower diffusion dynamics within the nanoporous network.

The differences observed between both sensing approaches cannot be attributed exclusively to the electrostatic or covalent nature of the functionalization strategy. Molecular dimensions, binding mechanisms, and the architecture of the functional layers formed within the nanopores also influence the optical response and binding kinetics. In the SpA/anti-IgG/IgG approach, the sequential immobilization of large biomolecules partially occupies the nanopore volume before IgG detection, which may affect molecular transport and binding-site accessibility. In contrast, APTES/GTA functionalization forms a smaller surface layer prior to LDH introduction. Therefore, the differences in saturation amplitude and characteristic response time arise from the combined effect of porosity, surface chemistry, molecular size, and binding mechanism. In the case of IgG detection (150 kDa), the relatively large molecules in the previous functionalization steps—namely SpA (42 kDa) and anti-IgG (150 kDa)—partially occupy the nanopore volume, which may hinder the diffusion of IgG molecules through the pores. In contrast, LDH detection involves a much smaller surface functionalization layer, as the GTA molecules are considerably smaller than SpA and anti-IgG. As a result, LDH molecules (150 kDa) can diffuse more easily within the nanoporous structure, leading to higher diffusion constants and higher characteristic times (*τ*).

## 4. Conclusions

This work investigates the influence of porosity and surface functionalization strategies on the biosensing performance of nanoporous anodic alumina rugate filters (NAA-RFs) using reflectometric interference spectroscopy (RIfS). The sensing response of NAA-RF structures was systematically analyzed as a function of porosity, which was controlled through the pore-widening process. In addition, two different surface chemistry approaches were evaluated to demonstrate the versatility of these nanostructures for selective biomolecule detection.

Structural characterization confirmed the formation of highly ordered nanoporous surfaces with a modulated pore diameter along the depth of the films. The controlled variation of pore widening enabled precise tuning of pore size and porosity while preserving the periodic modulation profile. Optical characterization revealed well-defined interferometric responses, including Fabry–Pérot oscillations and characteristic stop bands, whose spectral positions shifted as a function of porosity due to changes in the effective refractive index of the structures.

The sensing performance was evaluated using two different functionalization strategies. A sequential electrostatic approach based on SpA and anti-IgG was employed for IgG detection, while a covalent immobilization strategy using APTES and glutaraldehyde (GTA) was used for LDH detection. The results demonstrate that increasing porosity leads to enhanced EOT responses for both analytes, which could be attributed to the larger effective surface area available for molecular binding within the nanopores.

Kinetic analysis further revealed that while the saturation amplitude increases with porosity, the characteristic response time (*τ*) also increases at higher porosities, indicating slower molecular transport within the nanoporous network. This effect is attributed to diffusion limitations in highly porous structures, as well as differences in the surface functionalization layers and molecular interactions for each analyte.

Overall, this study highlights the importance of simultaneously tailoring structural parameters and surface chemistry in NAA-based nanostructures to optimize sensing performance. The results demonstrate the potential of modulated nanoporous architectures as versatile platforms for interferometric biosensing, enabling enhanced sensitivity and tunable detection of different biomolecules.

## Author contributions

J. M. C.: methodology, investigation, visualization, data curation, writing—original draft. P. F.: methodology, investigation, visualization, supervision, resources, formal analysis, writing—review and editing. J. F.-B.: resources, software, formal analysis, writing—review and editing. A. B.: writing—review and editing, formal analysis, supervision. L. F. M.: conceptualization, investigation, writing—review and editing, supervision, funding acquisition, formal analysis, methodology, project administration, resources. All authors have read and agreed to the published version of the manuscript.

## Conflicts of interest

There are no conflicts of interest to declare.

## Supplementary Material

NA-OLF-D6NA00533K-s001

## Data Availability

The data described in this manuscript are original and are not currently considered for publishing elsewhere. Supplementary information (SI): containing information about morphological parameters of NAA-RF, the kinetic fittings for different porosities and the equation for porosity estimation. See DOI: https://doi.org/10.1039/d6na00533k.
