# Supplementary material for: Tailoring porosity and surface functionalization in nanoporous anodic alumina rugate filters for enhanced interferometric biosensing
Source: Nanoscale Adv. 2026 Sep 2;8(21):6243–53. doi: 10.1039/d6na00533k (PMC13595583; doi:10.1039/d6na00533k)
Supplement: Supplementary file 1 [file NA-008-D6NA00533K-s001.pdf]

Supplementary Information (SI) for Nanoscale Advances.
This journal is © The Royal Society of Chemistry 2026

Supporting Information

Tailoring Porosity and Surface Functionalization in Nanoporous Anodic Alumina Rugate Filters for Enhanced Interferometric Biosensing

Josep Maria Cantons[1], Pilar Formentin[1], Josep Ferré-Borrull[1], Akash Bachhuka[2], Lluis F Marsal[1,*]

[1]Department of Electronic, Electric and Automatics Engineering, Rovira i Virgili University, Tarragona 43007, Spain

[2]Institute of Chemical Research of Catalonia (ICIQ), 43007 Tarragona, Spain

*E-mail: lluis.marsal@urv.cat

Pore diameter	Porosity (%)
28	7 ± 2
32	14 ± 2
42	18 ± 3
48	23 ± 3
50	32 ± 2
54	42 ± 4
57	52 ± 3
72	62 ± 4
88	70 ± 4

Figure S1: Estimation of porosity given the pore diameter and the optical measurements through RIfS.

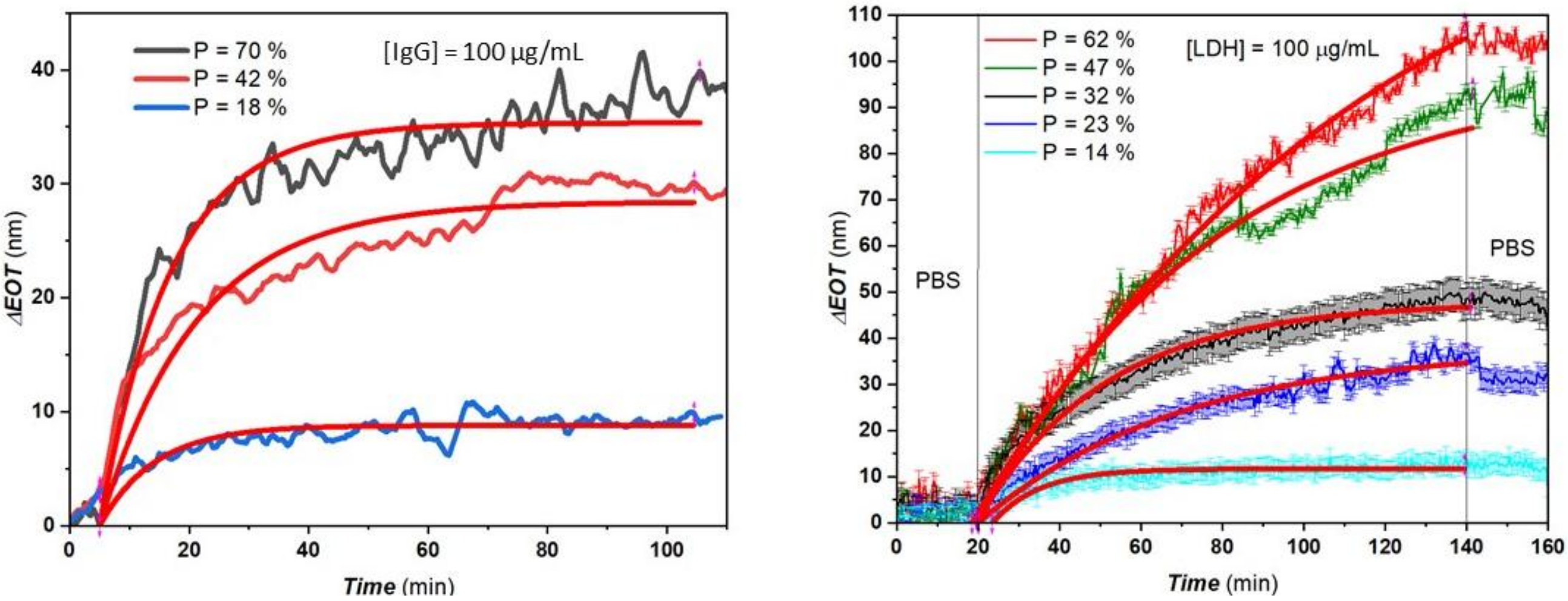

Figure S2: Kinetic fitting for IgG (left) and LDH (right) for different porosities.

$$P = \frac{\left(\frac{\mathrm{EOT}}{2L}\right)^{2/3} - n_{\mathrm{Al_2O_3}}^{2/3}}{n_{\mathrm{med}}^{2/3} - n_{\mathrm{Al_2O_3}}^{2/3}}$$

Equation S1. Porosity estimation based on the effective optical thickness (EOT) and the Looyenga effective medium approximation, where L is the physical thickness of the NAA-RF structure, and n_{Al2O3} and n_{med} are the refractive indices of alumina and the medium filling the nanopores, respectively.
